# Alterations in the carnitine cycle in a mouse model of Rett syndrome

**DOI:** 10.1038/srep41824

**Published:** 2017-02-02

**Authors:** Sabrina Mucerino, Anna Di Salle, Nicola Alessio, Sabrina Margarucci, Raffaella Nicolai, Mariarosa A. B. Melone, Umberto Galderisi, Gianfranco Peluso

**Affiliations:** 1 Institute of Bioscience and BioResources, CNR, Naples, Italy; 2 Department of Experimental Medicine, Biotechnology and Molecular Biology Section, University of Campania “Luigi Vanvitelli”, Naples, Italy; 3 Sigma-tau S.p.A., Pomezia, Rome, Italy; 4 Department of Medical, Surgical, Neurological, Metabolic Sciences, and Aging; Division of Neurology and InterUniversity Center for Research in Neurosciences, University of Campania “Luigi Vanvitelli”, Naples, Italy

## Abstract

Rett syndrome (RTT) is a neurodevelopmental disease that leads to intellectual deficit, motor disability, epilepsy and increased risk of sudden death. Although in up to 95% of cases this disease is caused by *de novo* loss-of-function mutations in the X-linked methyl-CpG binding protein 2 gene, it is a multisystem disease associated also with mitochondrial metabolic imbalance. In addition, the presence of long QT intervals (LQT) on the patients’ electrocardiograms has been associated with the development of ventricular tachyarrhythmias and sudden death. In the attempt to shed light on the mechanism underlying heart failure in RTT, we investigated the contribution of the carnitine cycle to the onset of mitochondrial dysfunction in the cardiac tissues of two subgroups of RTT mice, namely *Mecp2*^+/−^ NQTc and *Mecp2*^+/−^ LQTc mice, that have a normal and an LQT interval, respectively. We found that carnitine palmitoyltransferase 1 A/B and carnitine acylcarnitine translocase were significantly upregulated at mRNA and protein level in the heart of *Mecp2*^+/−^ mice. Moreover, the carnitine system was imbalanced in *Mecp2*^+/−^ LQTc mice due to decreased carnitine acylcarnitine transferase expression. By causing accumulation of intramitochondrial acylcarnitines, this imbalance exacerbated incomplete fatty acid oxidation, which, in turn, could contribute to mitochondrial overload and sudden death.

Rett syndrome (RTT) (OMIM ID: 312750) is a severe X-linked dominant neurological disorder, mainly affecting young females, that is the second most common cause of intellectual disability in women after Down syndrome^1^. In up to 95% of cases, this autism spectrum disorder is caused by *de novo* loss-of-function mutations in the gene mapped in human chromosome Xq28 that encodes the transcriptional regulator methyl-CpG-binding protein 2 (MeCP2)^2^,^3^. RTT is characterized by early neurological regression (starting during the first 6–16 months of life). It severely affects motor, cognitive and communication skills, and often leads to deceleration of head growth, autistic features and stereotyped hand movements^1^. The life expectancy of RTT patients is reduced and the mortality rate is 1.2% per annum; 26% of deaths are sudden and unexpected (mortality is 300-fold higher in RTT patients than in the general population)^4^. Epilepsy, brainstem autonomic failure, and respiratory failure/apnea have been implicated in sudden death in RTT^5^,^6^. Some RTT patients show prolonged QT intervals (LQT) on electrocardiograms, which is an electrophysiological condition that predisposes to the development of ventricular tachyarrhythmias and sudden death^7^. LQTs are present also in a subgroup of the murine model of RTT^8^.

The cause of LQT in RTT is unknown, although several studies suggested that it may be related to mitochondrial dysfunctions^9^,^10^,^11^,^12^. This concept is in line with the finding that mice with an *Mecp2* null allele develop a metabolic syndrome characterized by insulin resistance, hypertriglyceridemia, obesity^13^, and altered cholesterol metabolism^14^, all of which are signs of mitochondrial metabolic imbalance. Moreover, an excess of unprocessed fatty acids and an abnormal carnitine profile have been identified in the serum of children affected by RTT but not in controls^15^, which suggests an interplay among mitochondrial function, energy metabolism and fatty acid oxidation (FAO). In this context, the therapeutic potential of carnitine or carnitine derivatives has been evaluated in RTT mouse models and in clinical trials^16^,^17^,^18^. In particular, treatment of RTT patients with acetyl-L-carnitine and L-carnitine improved sleep, energy level, communication, cardiac function, and neuronal morphology in the hippocampus^17^. Acetyl-L-carnitine also improved a number of electrocardiographic parameters in RTT patients^19^ thereby reducing the risk of cardiac arrhythmias and mitigating the risk of sudden death. Given the low plasma level of L-carnitine in some RTT patients^18^ and in patients with other autism spectrum disorders^20^, carnitine deficiency has been implicated in the pathogenesis of heart dysfunction in RTT patients. However, Schaevitz *et al*.^17^. found no significant differences in plasma carnitine levels between control and RTT mice although they did find that carnitine treatment prevented metabolic abnormalities and improved cognitive function in *Mecp2*-null mice. These conflicting results are compatible with the concept that it is a perturbation of the carnitine system rather than a carnitine deficiency *per se* that is involved in the pathogenesis of heart failure in RTT patients.

Mitochondrial FAO is the major metabolic pathway for producing both ATP, NADH and NADPH^21^,^22^, and needs the carnitine system to import fatty acids into the mitochondria. The carnitine cycle requires L-carnitine and is composed of two acyltransferases, carnitine palmitoyltransferases 1 and 2 (CPT1 and CPT2), and carnitine acylcarnitine translocase (CACT), which is a mitochondrial carrier (aka SLC25A20)^23^,^24^,^25^. CPT1 catalyzes the rate-limiting reaction in the transesterification of acyl-CoA to acylcarnitine. CACT transports acylcarnitines across the inner mitochondrial membrane in exchange for a free carnitine molecule. CPT2 completes the cycle in the mitochondrial matrix by reconverting acylcarnitine into an acyl-CoA. Although involved mainly in mitochondrial acyl-CoA import, CPT2 and CACT can operate in the reverse direction in case of an increase in intramitochondrial acyl-CoA levels. Also carnitine acetylcarnitine transferase (CRAT), which is involved mainly in the formation of short-chain acylcarnitines, cooperates with CACT in decreasing the intramitochondrial acyl-CoA/CoA ratio^23^,^26^. This mechanism facilitates the export of FAO intermediates as acylcarnitines from the mitochondria to the cytosol.

In the attempt to shed light on the mechanism underlying heart failure in RTT, we investigated the contribution of the carnitine cycle to the onset of mitochondrial dysfunction in RTT cardiac tissue using an *in vivo* mouse model. Here we provide evidence that acylcarnitines and components of the carnitine system orchestrate the interplay between the metabolism of fatty acids in the cardiac mitochondria and heart failure in RTT patients. Indeed, the upregulation (in terms of expression and activity) of mitochondrial CPT1, CACT and CPT2 associated with downregulation of CRAT found in the LQT RTT subgroup of mice could impair metabolic flexibility, which is required for maintenance of normal cardiac function.

## Results

### Grouping of RTT mice

An LQT in electrocardiograms, which reflects abnormal cardiac phenotypes^8^,^27^,^28^, is a frequent feature of RTT patients and in RTT mice models. In a preliminary study conducted to determine the age at which to perform metabolic analyses, we recorded surface ECGs from birth to 18 months in female RTT mice (B6.129P2(C)-Mecp2tm1.1Bird/J, designated *Mecp2*^+/−^), and found that, compared with WT mice, significantly more 7-month-old *Mecp2*^+/−^ mice had LQT intervals. This percentage increased to ~37% in 11-month-old mice. No ECG abnormalities were found in WT mice. Representative baseline ECGs are shown in Supplementary Fig. S1. We then grouped *Mecp2*^+/−^ mice on the basis of their QT interval, and euthanized them at 11 months of age. As shown in Table 1, *Mecp2*^+/−^ NQTc mice exhibited QTc values between 380 and 410 ms (mean: 401 ms), while the corrected QT interval of *Mecp2*^+/−^ LQTc mice ranged between 460 and 550 ms (mean: 480 ms), which is significantly higher than that recorded in WT and *Mecp2*^+/−^ NQTc mice (*P* < 0.001).

### Analysis of the carnitine cycle

**Figure 1:**
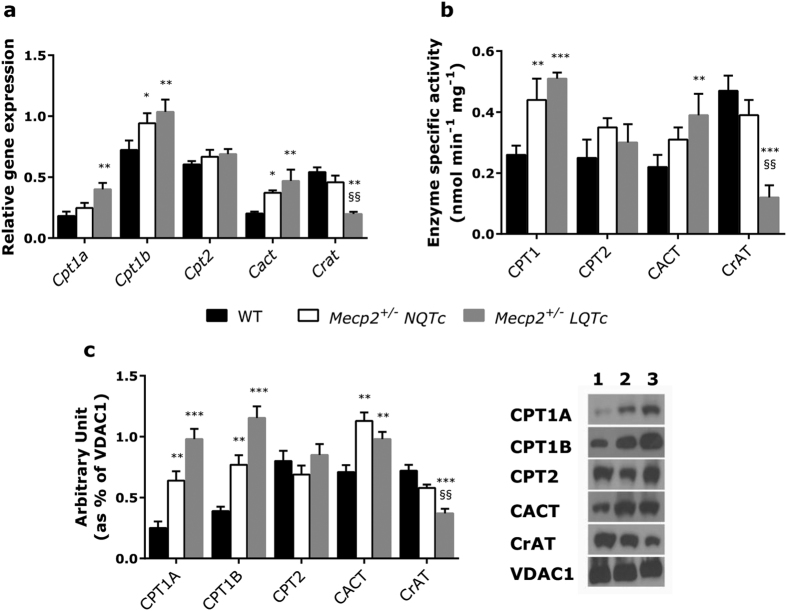
mRNA and protein expression of the carnitine system in heart tissues of WT (n = 15), *Mecp2*^*+/−*^ NQTc (n = 30) and *Mecp2*^*+/−*^ LQTc (n = 10) mice. (**a**) qRT-PCR of *Cpt1a, Cpt1b, Cpt2, Cact* and *Crat*. The target gene expression was normalized to the housekeeping genes *Hprt1* and *Actb*. Relative differences in PCR results were calculated using the comparative cycle threshold method (2^−ΔΔCT^). (**b**) The enzymatic activities were determined in mitochondrial fractions purified from heart tissues of WT, *Mecp2*^+/−^ NQTc, and *Mecp2*^+/−^ LQTc mice as described in Experimental procedures section. (**c**) Western blot analysis of proteins CPT1A, CPT1B, CPT2, CACT and CrAT in the mitochondrial enriched fraction. The expression of each protein was normalized to the housekeeping protein VDAC1. Representative cropped bands of the WB gel blots are 1: WT; 2: *Mecp2*^+/−^ NQTc; 3: *Mecp2*^+/−^ LQTc. Uncropped western blots were provided in Supplementary Fig. S2. For each mitochondrial preparation, three different experiments were conducted and the results expressed as the mean of the values obtained (mean ± SD). The bars represent means ±standard deviation. Statistically significant variations: *P < 0.05 *versus* WT; **P < 0.01 *versus* WT; ***P < 0.001 *versus* WT; ^§§^*P* < 0.01 *versus Mecp2*^+/−^ NQTc.

The heart’s continuous contractile activity is sustained primarily via β-oxidation of long-chain fatty acids^27^. Therefore, to determine if the mitochondrial dysfunction in heart tissue might involve the carnitine system, we investigated Cpt1a, Cpt1b, Cpt2, Cact and CrAT expression and functionality in the explanted heart of *Mecp2*^+/−^ NQTc and *Mecp2*^+/−^ LQTc mice. As shown in Fig. 1a and c, qRT-PCR and Western blot analysis revealed significantly higher Cpt1a, Cpt1b and Cact levels in *Mecp2*^+/−^ NQTc and *Mecp2*^+/−^ LQTc mice versus WT, and the statistical significance was greater in *Mecp2*^+/−^ LQTc mice. Surprisingly, the level of the Crat gene and protein was significantly lower in *Mecp2*^+/−^ LQTc than in WT and *Mecp2*^+/−^ NQTc mice. In addition, *Cpt1a* expression was lower than *Cpt1b* expression in the cardiac tissues of all mice examined (Fig. 1a), in agreement with the finding that CPT1B protein expression was higher than the isoform A in the post-neonatal heart of mice and humans^28^.

The analysis of enzyme activity on mitochondrial enriched fractions isolated from heart tissues of *Mecp2*^+/−^ NQTc and *Mecp2*^+/−^ LQTc mice confirmed an increase in CPT1 and CACT and a loss of function of CrAT activity compared to WT mice (Fig. 1b). In particular, CPT1 activity was 70% higher and CACT activity 41% higher in *Mecp2*^+/−^ NQTc mice than in WT mice. In *Mecp2*^+/−^ LQTc mice, CPT and CACT activities increased by 96% and 77%, respectively versus WT mice. Conversely, CrAT activity was significantly lower ( > 70%) in *Mecp2*^+/−^ LQTc mice than in either *Mecp2*^+/−^ NQTc or WT mice (*P* < 0.01 *versus Mecp2*^+/−^ NQTc and *P* < 0.001 *versus* WT).

### Acylcarnitine profile

**Figure 2:**
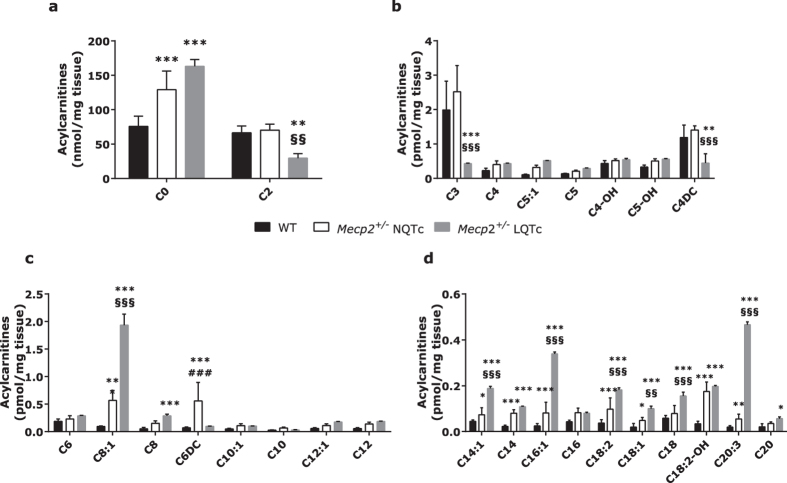
Free carnitine and acylcarnitine profiling in heart tissues of WT (n = 15), *Mecp2*^+/−^ NQTc (n = 30) and Mecp2^+/−^ LQTc (n = 10) mice. The acyl chain length (C) is denoted by the corresponding metabolite number (e.g., C0 = free carnitine, C2 = acetylcarnitine; C3 = propionylcarnitine). (**a**) C0 and C2 levels; (**b**) short-chain, (**c**) medium-chain and (**d**) long-chain acylcarnitines. Data are expressed as mean ± SD. Statistically significant variations: *P < 0.05 *versus* WT; **P < 0.01 *versus* WT; ***P < 0.001 *versus* WT; ^§§^*P* < 0.01 *versus Mecp2*^+/−^ NQTc; ^§§§^*P* < 0.001 *versus Mecp2*^+/−^ NQTc; ^###^*P* < 0.001 *versus Mecp2*^+/−^ LQTc.

To verify if perturbation of the carnitine system could result in accumulation of toxic lipid intermediates, we evaluated the acylcarnitine profile in *Mecp2*^+/−^ NQTc and *Mecp2*^+/−^ LQTc heart tissue extracts. We used tandem mass spectrometry to analyze free carnitine (C0) and 55 independent acylcarnitine species ranging in size from 2 to 24 carbons (see Supplementary Fig. S2). This analysis provides a realistic snapshot of mitochondrial efficiency and is commonly used to detect metabolic dysfunctions^29^. Acetylcarnitine (C2) and propionylcarnitine (C3) levels (Fig. 2a,b) were significantly lower in *Mecp2*^+/−^ LQTc mice than in WT animals (*P* < 0.01 and *P* < 0.001, respectively), in accordance with the decrease observed in CrAT activity. In addition, several medium- and long-chain acylcarnitines were more abundant in *Mecp2*^+/−^ LQTc mice than in WT mice (Fig. 2b–d).

### Acetyl-CoA content and PDH activity

**Figure 3:**
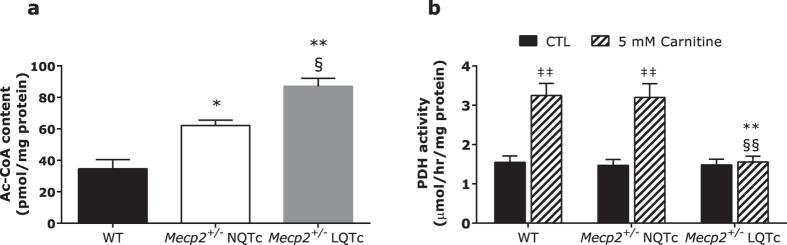
Acetyl-CoA content and PDH activity in mitochondrial fractions of mice heart tissues. (**a**) Acetyl-CoA levels were determined as described and values normalized to the mitochondrial protein content. Data are expressed as mean ± SD. (**b**) PDH activity was determined by measuring ^14^CO_2_ produced from 1 mM [1-^14^C]pyruvate ± 5 mM carnitine in mitochondria enriched fraction from RTT and WT mice. PDH activity in absence of carnitine was used as control (CTL). All values were normalized to the mitochondrial protein content and data expressed as mean ± SD. *P < 0.05 *versus* WT; **P < 0.01 *versus* WT; ^§^*P* < 0.05 *versus Mecp2*^+/−^ NQTc; ^§§^*P* < 0.01 *versus Mecp2*^+/−^ NQTc; ^‡‡^*P* < 0.01 *versus* CTL.

Acetyl-CoA is a potent allosteric inhibitor of glycolysis and pyruvate dehydrogenase (PDH) in the regulation of the homeostatic mechanism that controls circulating concentrations of glucose and fatty acids (Randle cycle)^30^. Given that CrAT lowers acetyl-CoA and regenerates free CoA, impairment of CrAT function can increase the mitochondrial acetyl-CoA level, which in turn induces glucose oxidation inhibition via PDH phosphorylation. As shown in Fig. 3, concentrations of mitochondrial acetyl-CoA were significantly higher in both *Mecp2*^+/−^ NQTc and *Mecp2*^*+/−*^LQTc mice than in WT mice. Acetyl-CoA accumulation was more evident in *Mecp2*^+/−^ LQTc mice than in either *Mecp2*^+/−^ NQTc or WT mice (*P* < 0.05 and *P* < 0.01, respectively).

To verify whether the increase in mitochondrial acetyl-CoA level inhibited PDH activity, we measured mitochondrial PDH activity in the presence and absence of carnitine. As shown in Fig. 3b, PDH activity was significantly higher (about 2-fold, *P* < 0.01) in WT and *Mecp2*^+/−^ NQTc mice treated with carnitine than in untreated mice, whereas PDH activity remained unchanged in *Mecp2*^+/−^ LQTc mice. These results suggest that CrAT deficiency in *Mecp2*^+/−^ LQTc mice could result in persistent PDH inhibition also in the presence of carnitine.

### Substrate oxidation

**Figure 4:**
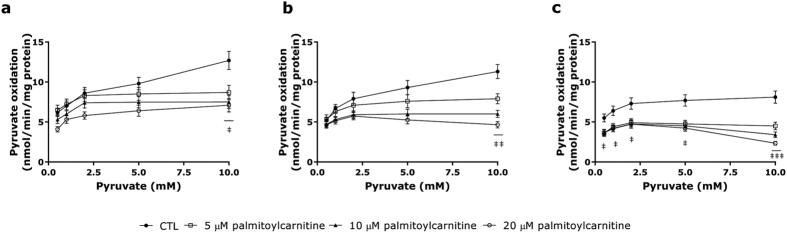
Pyruvate oxidation. Determination of pyruvate (0.5, 1, 2, 5, and 10 mM) oxidation in presence of an increasing concentration of palmitoylcarnitine in mitochondrial enriched fractions from (**a**) WT, (**b**) *Mecp2*^+/−^ NQTc, and (**c**) *Mecp2*^+/−^ LQTc. Pyruvate oxidation in absence of palmitoylcarnitine was used as control (CTL). All values were normalized to the mitochondrial protein content and data expressed as mean ± SD. ^‡^*P* < 0.05 *versus* CTL; ^‡‡^*P* < 0.01 *versus* CTL; ^‡‡‡^*P* < 0.001 *versus* CTL.

Measurement of pyruvate and palmitate oxidation in mitochondria can reveal how pyruvate and fatty acids compete for energy production pathways in *Mecp2*^+/−^ compared to WT mice. The measurement of pyruvate oxidation provides an index of tricarboxylic acid cycle function in response to different palmitoylcarnitine concentrations. As shown in Fig. 4, in the absence of long-chain fatty acids, pyruvate oxidation was similar in the three experimental groups, although pyruvate oxidation capacity was slightly reduced in *Mecp2*^+/−^ LQTc mice at the highest pyruvate concentration used. Palmitoylcarnitine supplementation inhibited pyruvate oxidation in all groups, although this effect was significantly (*P* < 0.01) more pronounced in *Mecp2*^+/−^ LQTc mice versus controls, whereas 5 μM and 20 μM palmitoylcarnitine produced the same effect in WT mice already in the presence of 0.5 mM pyruvate.

**Figure 5:**
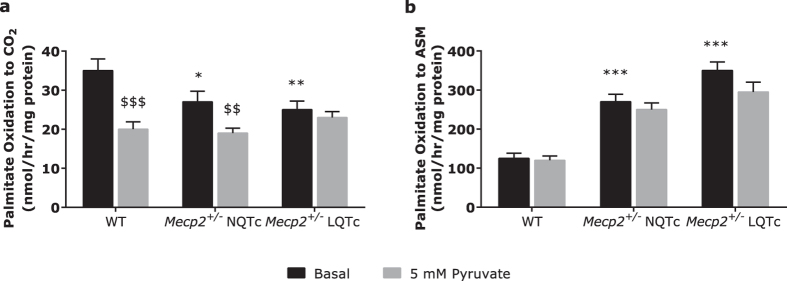
Analysis of fatty acid oxidation. Oxidation of [1-^14^C] palmitate to ^14^CO_2_ (**a**) or ^14^C-labeled acid soluble metabolites (ASMs; **b**) was measured in mitochondrial enriched fractions in absence or in presence of pyruvate as an index of substrate switching. All values were normalized to the mitochondrial protein content and data expressed as mean ± SD. ^$$^*P* < 0.01 pyruvate-induced inhibition of palmitate oxidation *versus* basal oxidation; ^$$$^*P* < 0.001 pyruvate-induced inhibition of palmitate oxidation *versus* basal oxidation; *P < 0.05 *versus* WT; **P < 0.01 *versus* WT.

To evaluate metabolic inflexibility in *Mecp2*^+/−^ mice, we determined the dose-dependent pyruvate inhibition of fat oxidation as both complete (CO_2_ production, Fig. 5a) and incomplete (acid soluble metabolite [ASM] production; Fig. 5b) palmitate oxidation. In the absence of pyruvate (basal condition), mitochondrial fatty acid oxidation to CO_2_ was lower, and incomplete oxidation to ASMs was higher in *Mecp2*^+/−^ NQTc and *Mecp2*^+/−^ LQTc mice than in WT mice. In detail, *Mecp2*^+/−^ LQTc mice exhibited a significant decline in complete palmitate oxidation to CO_2_ (*P* < 0.001 versus WT) and a significant increase in incomplete oxidation to ASMs (*P* < 0.001 versus WT), which indicates a remarkable imbalance in the ratio of incomplete to complete fat oxidation. In addition, in WT and *Mecp2*^+/−^ NQTc mice, 5 mM pyruvate induced a decrease in palmitate oxidation to CO_2_, which is a sign of correct substrate switching, whereas the pyruvate-mediated inhibition of fat oxidation was abolished in *Mecp2*^+/−^ LQTc mice. Taken together these results indicate that *Mecp2*^+/−^ LQTc mice were unable to switch their metabolism correctly according to substrate availability, thereby leading to mitochondrial dysfunction and imbalance.

## Discussion

Here we demonstrate that mitochondrial dysfunction is related to perturbation of the carnitine cycle in the heart muscle of a mouse model of RTT. The link between mutations in the *Mecp2* gene and mitochondrial dysfunction is unclear, although such clinical signs as hypotonia^31^ and myocardial dysfunction^32^ correlate with mitochondrial dysfunction^33^,^34^,^35^,^36^. In addition to ultrastructural alterations of mitochondria (i.e., vacuolization, granular inclusions and membranous changes)^37^,^38^, a decrease in NADH cytochrome c reductase, succinate cytochrome c reductase, and cytochrome c oxidase activity^31^,^37^ has been reported in muscle^39^ and brain biopsies^40^,^41^ of RTT patients and of the RTT mouse model. Recently, an association has been suggested between FAO deficiencies and developmental brain disorders such as autism spectrum disorders, to which RTT belongs^42^. The altered circulating levels of carnitine and/or acyl-carnitine (i.e., phenotypes suggestive of deficiencies in the long-chain FAO) present in RTT children would support this hypothesis^20^,^32^. As a consequence of these mitochondrial alterations, mitochondrial energy production would be less efficient and lead to reduced ATP levels, as detected in a magnetic resonance study on *Mecp2*-deficient mice^10^. Although these data are indicative of widespread mitochondrial dysfunction, the cause of defective mitochondria is unclear, and it remains to be determined whether and how alterations in the mitochondrial metabolic pathways are involved in the pathogenesis of autism spectrum disorders and cause functional impairment of organs, as in the case of cardiac dysfunction observed in both RTT patients and animal models^8^,^32^,^43^.

We evaluated carnitine-dependent mitochondrial metabolic pathways in two subgroups of 11-month-old RTT mice: *Mecp2*^+/−^ NQTc mice that have a normal QT interval, and *Mecp2*^+/−^ LQTc mice that have an LQT interval. We carried out these experiments on explanted hearts to evaluate whether alterations of the carnitine profile and the carnitine cycle were present at tissue level in either of these two conditions. In addition, the two groups of mice did not differ in terms of body weight, insulin resistance or O_2_ consumption/CO_2_ expiration (see Supplementary Table S2 and Fig. S4). Again, the cardiac ATP level practically overlapped in the two groups of animals, which suggests that alteration of the carnitine cycle has a relevance that goes beyond the energetic aspect (see Supplementary Table S3).

To our knowledge, this is the first study to demonstrate significantly upregulated expression of CPT1A/B and CACT at mRNA and protein level in the heart of *Mecp2*^+/−^ mice. Interestingly, the increase in these proteins has been linked to high rates of incomplete fat oxidation and intracardiac accumulation of fatty acylcarnitines, which are byproducts of lipid catabolism, in both groups of *Mecp2*^+/−^ mice. The high level of myocardial acylcarnitines induced by increased CPT1 activity might indicate that the rate of fatty acid catabolism exceeds the flux through the tricarboxylic acid cycle. In this case, the accumulating intramitochondrial acyl-CoA intermediates are converted back to acylcarnitines, which can then exit the organelle and tissue via CACT. Indeed, in addition to its role in fatty acid oxidation, carnitine enables mitochondrial export of excess carbon fuel, thereby buffering intramitochondrial imbalances between the acyl-CoA load and Krebs cycle activity. Here we show that the protein level and activity of CrAT were significantly reduced only in the *Mecp2*^+/−^ LQTc mice. CrAT resides in the mitochondrial matrix and converts acetyl-CoA, which derives from fatty acid, glucose and amino acid catabolism, to membrane permeant acetylcarnitine. Thus, CrAT facilitates the trafficking and efflux of short-chain acyl-CoA intermediates from the mitochondrial compartment to other cellular and extracellular sites.

It is now recognized that the carnitine cycle and CrAT play important roles in regulating metabolic flexibility and mitochondrial substrate switching in mouse skeletal muscle^44^,^45^. Moreover, several findings indicate an interplay between CrAT and PDH. First, acetyl-CoA antagonizes PDH activity by directly and indirectly inhibiting the enzyme complex, while carnitine treatment, which increases tissue acetylcarnitine efflux, stimulates the PDH complex in human muscle mitochondria^44^,^45^,^46^. Second, muscle-specific deletion of CrAT in mice and human myocytes decreases glucose tolerance and metabolic flexibility^45^. Third, insulin resistance and lipid stress inhibit PDH and CrAT in muscle mitochondria thereby increasing susceptibility to mitochondrial dysfunction^44^. Our finding that inhibition of pyruvate oxidation by palmitoylcarnitine was significantly more pronounced in heart mitochondria from the *Mecp2*^+/−^ LQTc group reinforces the role played by CrAT in myocardium metabolism. Moreover, the difference between genotypes was amplified by the addition of pyruvate, which inhibited palmitate oxidation more robustly in the *Mecp2*^+/−^ LQTc group. In aggregate, our findings established that CrAT deficiency resulted in abnormal fuel selection and a corresponding decline in metabolic flexibility.

Cardiac metabolism is primarily aerobic, with 60 to 90% of heart ATP produced via mitochondrial oxidation of long-chain fatty acids whereas the remaining 10% to 40% derives from oxidation of glucose and lactate, and from small amounts of ketone bodies and certain amino acids^47^,^48^. The adult heart can switch energy sources according to metabolic demand, oxygen, and substrate availability, as in the Randle cycle^30^,^49^. The switch towards glucose metabolism improves myocardial contractile efficiency by increasing the stoichiometric ratio of ATP production to oxygen consumption, besides minimizing oxidative losses through mitochondrial respiratory chain uncoupling associated with free fatty acid metabolism. Thus, the failure to maintain the metabolic flexibility of the heart is closely related to cardiovascular disease and heart failure^50^,^51^,^52^,^53^. Remodeling of cardiac metabolism in RTT mice, characterized by abnormally high myocardial dependence on fatty acid metabolism, has been reported in animal and human studies of metabolic syndrome, insulin-resistance or high adrenergic states; the derangements most often-cited being enhanced fatty acid oxidation and impaired glucose utilization^54^. A loss of *Mecp2* contributes to metabolic syndrome by increasing insulin secretion and decreasing insulin signaling – a process that leads to a complete dissociation between insulin levels and the appropriate metabolic effects of insulin on glucose regulation^55^.

It remains to be clarified why CrAT expression decreases with aging in RTT patients and whether or not this decrease is associated with the *Mecp2* mutation. In this context, carnitine supplementation to RTT mice might mitigate this mitochondrial stress by shunting surplus acetyl-CoA towards the CrAT reaction. Indeed, although the release of acetylcarnitine from the heart is a valve for acetyl production, the flow through this valve is limited by the cardiac pool of carnitine. It is noteworthy, however, that in case of CrAT deficiency, carnitine supplementation might not exert therapeutic effects^16^,^18^,^19^.

Here we demonstrate that the carnitine cycle plays a central role in shuttling carbon fuels between cell compartments associated with *de facto* regulation of mitochondrial and intracellular trafficking of acetyl- and other reactive acyl-CoA moieties. Indeed, the imbalanced carnitine system in *Mecp2*^+/−^ LQTc mice exacerbates incomplete FAO, which accumulates intra-mitochondrial acylcarnitines that could contribute to mitochondrial overload. In conclusion, our data suggest that the simultaneous presence of a long QT interval and the onset of metabolic inflexibility may be the cause of sudden death in RTT.

## Experimental procedures

### Animals

MeCP2^tm1.1Bird+/−^ female mice (*Mecp2*^+/−^) were purchased from the Jackson Laboratory (ME, USA) (Jax stock number: 003890) and mated with C57BL/6 wild-type male mice (Jax stock number: 000664). Wild-type female littermates served as controls (WT Eleven-month-old WT and RTT mice were euthanized via cervical dislocation under anesthesia, and hearts were rapidly dissected, washed in 1 × PBS to remove blood, and snap frozen in liquid nitrogen. National or institutional guidelines were applied in the care and use of animals, and all experimental protocols were approved by the Ethics Committee of the Second University of Naples.

### Genotyping

Genomic DNA was extracted from tail tips. The genotype of the mice was determined by polymerase chain reaction (PCR) using PCR primers as described on the web site of Jackson Laboratories.

### Surface electrocardiogram (ECG)

Over a period of 18-months a cohort of *Mecp2*^+/−^ mice were monitored every two months by surface ECG, starting from 1 month of age. A cohort of *Mecp2*^+/−^ mice underwent surface ECG recording every two months for 18 months starting from 1 month of age. Mice were anesthetized with 1.5% isoflurane in 95% O_2_ and six-lead ECGs were recorded by pad electrodes with band-pass filtering between 0.03 Hz and 1 kHz, according to McCauley *et al*.^8^. QT values and corrected QT intervals (QTc) were calculated as described elsewhere^56^. In detail, QT represents the interval from the beginning of the Q wave to the end of the T wave, while QTc was calculated with the formula QTc = QT + 0.3173 × (170 − RR), where RR is the time interval between two consecutive RR waves, and 170 is the RR-interval at the reference rate of 350 b.p.m^57^. QT was considered normal if <450 milliseconds and prolonged if >450 ms. Eleven-month-old RTT mice underwent ECG euthanized and divided into 2 groups based on their QTc interval duration: normal QTc (*Mecp2*^+/−^ NQTc, n = 30) and long QTc (*Mecp2*^+/−^ LQTc, n = 10).

### Real-Time Polymerase Chain Reaction (qRT-PCR)

Total RNA was extracted from cardiac tissues using TRIzol reagent (Invitrogen, Italy) according to the manufacturer’s instructions. Total RNA (0.2 μg) was first treated at 37 °C for 30 min with DNase (RQ1 RNase-Free DNase – Promega, Italy) and then subjected to reverse transcription (RT) with 0.4 μg random hexamers and 20 U AMV reverse transcriptase (Promega) in a 25-μl reaction mixture at 42 °C for 1 h. The resulting mixture was amplified by qRT-PCR using specific primers for carnitine palmitoyltransferase 1 A (*Cpt1a*), carnitine palmitoyltransferase 1B (*Cpt1b*), carnitine palmitoyltransferase 2 (*Cpt2*), carnitine acylcarnitine translocase (*Cact*), carnitine acetylcarnitine transferase (*Crat*), hypoxanthine phospho-ribosyltransferase (*Hprt1*) and β-actin (*Actb*) as listed in Supplementary Table S1. Real-time PCR assays were run on an Opticon-4 machine (BioRad, Italy). The reactions were performed according to the manufacturer’s instructions using SYBR Green PCR Master mix (Invitrogen). The PCR conditions were: AmpliTaq Gold^®^ DNA Polymerase (Life Technologies, Italy) activation for 10 min at 95 °C and 40 cycles at 95 °C (denaturation) for 15 s and 60 °C (annealing/extension) for 1 min. All the reactions were run in triplicate and two housekeeping genes, *Hprt1* and *Actb* were used to normalize each sample. Relative differences in the qRT-PCR results were calculated using the comparative cycle threshold (2^−ΔΔCT^) method.

### Mitochondrial isolation

Cardiac tissues were homogenized and the mitochondria fraction isolated as previously reported^58^. The isolation procedure was performed at 4 °C. Briefly, cardiac tissues were resuspended in 10 mM Tris-HCl pH 7.8, 0.2 mM ethylenediaminetetraacetic acid (EDTA), 250 mM sucrose (Mito buffer) containing 100 mg/ml of complete Protease Inhibitor Cocktail Tablets (Complete Mini, Roche, Italy), and disrupted using a dounce with a tight-fitting Teflon pestle. Mitochondrial pellet (P) and supernatant (S) fractions were obtained by sequential centrifugation at 8000 g for 5 min, and 3500 g for 5 min. To obtain a pellet rich in mitochondria, the purified fractions were resuspended in Mito buffer without sucrose and subjected to further centrifugation in the presence of a sucrose gradient (1–1.5 M) at 100,000 g for 1 h. Protein content was estimated by the method of Bradford *et al*.^59^, using bovine serum albumin as standard.

To assess the functional integrity of the isolated mitochondria, we measured the changes in mitochondrial membrane potential (MMP, ΔΨm) using the 5,5′,6,6′-tetrachloro-1,1′,3,3′-tetraethyl benzimidazolyl carbocyanine iodide (JC-1) probe (1st J-aggregate-forming cationic dye) as previously described^60^. The mitochondrial-enriched fraction was treated with 200 μM JC-1 and incubated at 37 °C for 30 min. The samples were centrifuged at 800 g for 15 min and the pellets, resuspended in Mito buffer, were read on a fluorimeter (excitation filter 485 nm and emission filters 520 nm/595 nm). Carbonyl cyanide 3-chlorophenylhydrazone (CCCP), a mitochondrial depolarizing agent, was used as a positive control for MMP reduction. In addition, citrate synthase, which is a marker of mitochondrial content, was evaluated as reported by Andersen *et al*.^61^. Mitochondrial fractions selected for further experiments showed a percentage of integrity ranging between 78 and 95%, and were homogenous in terms of citrate synthase activity (see Supplementary Table S4).

### Western blotting

Sodium dodecyl sulfate polyacrylamide gel electrophoresis and immunoblotting were carried out according to standard procedures in triplicate using 30 μg of mitochondrial enriched fractions. Antibodies were mouse polyclonal anti–carnitine palmitoyltransferase 1 A (1:1000; Sigma-Aldrich), mouse polyclonal anti–carnitine palmitoyltransferase 1B (1:1000; Abcam, Italy), mouse monoclonal anti–carnitine palmitoyltransferase 2 (1:1000; Abcam), mouse polyclonal anti–carnitine acylcarnitine translocase (1:1000; Abcam), mouse polyclonal anti–carnitine acetylcarnitine transferase (1:1000; Santacruz Biotechnology, Italy), rabbit polyclonal anti-VDAC1/Porin (1:1000; Abcam). Anti-mouse or anti-rabbit were used as secondary antibodies (1:10.000). The relative expression, normalized in relation to the housekeeping protein (VDAC1), was quantified densitometrically using Quantity One^®^ 1-D analysis software (BioRad, Italy).

### Enzymatic determinations

CPT1 activity was assayed in mitochondria as the incorporation of radiolabeled carnitine into acylcarnitine according to Ricciardi *et al*.^62^. The reaction mixture contained, in a final volume of 0.5 ml: 30 mM KCl, 105 mM Tris-HCl, pH 7.2, 50 mM palmitoyl-CoA, 500 mM [^14^C]L-carnitine (~280,000 cpm), 1% fatty acid-free bovine serum albumin, 4 mM ATP, 4 mM MgCl_2_, 0.25 mM GSH, 2 mM KCN, 40 mg/ml rotenone, and 40 μg of mitochondrial proteins. To measure CPT activity the mitochondria were kept intact, and incubations were carried out in the absence or presence of 100 mM malonyl-CoA. CPT activity insensitive to 100 mM malonyl-CoA was always subtracted from the experimentally determined CPT activity. CPT 2 activity was measured using the same system except that the mitochondrial suspension was first brought to 1% (w/v) with octyl glucoside (OG) and kept on ice for 30 min before assay. This treatment destroys CPT 1 activity while causing complete release of the detergent-stable and malonyl-CoA-insensitive CPT 2 isoform^63^. Reactions were initiated by the addition of mitochondria (40 μg) and were terminated with 0.5 ml of 1.2 N HCl after 4 min. The palmitoyl-[^14^C]L-carnitine formed was extracted with butanol and quantified by scintillation counting^63^,^64^. CACT was assayed in mitochondrial fractions according to Peluso *et al*.^65^ and Ijlst *et al*.^66^. Briefly, the reaction was initiated by adding isolated mitochondria (40 μg) loaded with 2 mM L-[methyl-^14^C]-carnitine (specific activity ~7,200 cpm/mmol) to 0.5 ml of a solution containing 200 mM mannitol, 50 mM Tris-HCl (pH 7.4), and unlabeled carnitine or palmitoylcarnitine. Exchange rates were calculated as described by Paradies *et al*.^67^. CrAT activity was determined in accordance with Muoio *et al*.^45^. Isolated mitochondria (40 μg) were resuspended in Mito buffer and processed by freeze fracturing five times and sonication. The reaction mixture included 50 mM Tris-HCl, 1 mM EDTA, 0.45 mM acetyl-CoA and 0.1 mM 5,5′-dithiobis-2-nitrobenzoic acid (DTNB) at pH 7.8. Reactions were started with the addition of 5 mM L–carnitine, and the rate was monitored at 412 nm with a Cary 100 double-beam spectrophotometer (Agilent, Italy) following the reduction of DTNB (ε_mM_ for TNB = 16029 M^−1^ cm^−1^), and using a blank containing all reagents except carnitine. All enzymatic activities were determined in at least three different experiments performed in triplicate.

### Acyl-carnitine profile

Tissue samples were processed and analyzed by tandem mass spectrometry according to Muoio *et al*.^45^. Briefly, specimens of powdered cardiac muscle tissue were homogenized in 9 volumes of deionized water using a Potter-Everhelm Teflon-glass homogenizer and centrifuged at 14,000 rpm for 10 min at 4 °C. The supernatant (50 μl) was treated with 400 μl di acetonitrile and centrifuged at 2,000 g for 5 min. The supernatant (200 μl) was evaporated using a Savant™ SpeedVac™ (Thermo Scientific, Italy) Concentrator. The residues were incubated with either 3 M MeOH-HCl at 50 °C for 15 min or 3 M BuOH-HCl at 65 °C for 15 min, depending on whether methyl ester or butyl ester derivatives of the acylcarnitines were to be prepared. The reagent was evaporated and 100 μl methanol:water (85:15, v/v) was added. Samples were shipped to the Bambin Gesù hospital facility on dry-ice and stored there at −80 °C until analysis.

### Acetyl-CoA measurement

The concentration of acetyl-CoA was evaluated in the mitochondrial fraction after sonication by fluorescence assay using a commercial kit according to the manufacturer’s instructions (Sigma-Aldrich, Italy). Values were expressed as acetyl-CoA pmol and were normalized to the mitochondrial protein content (mean ± SD).

### Energy substrate oxidation

To eliminate internal acylcarnitines, the mitochondria were incubated at 37 °C in the presence of 15 mM carnitine for 15 minutes, and then washed twice with Mito buffer. Pyruvate oxidation was measured in a reaction mixture containing 1 mM NAD, 5 mM ADP, 100 μM CoA, 0.5 mM malate and various concentrations of pyruvate (0.5, 1, 2, 5 or 10 mM, non-labeled pyruvate and 01 μCi/ml of [1-^14^C]pyruvate, PerkinElmer, Italy) in the presence or absence of palmitoylcarnitine (5, 10 or 20 μM). ^14^CO_2_ produced during the enzymatic reaction was trapped in benzethonium chloride-saturated filter paper placed in a tube stopper. After 1 h, the reaction was stopped by adding 25% HClO_4_ and the ^14^CO_2_ was quantified by scintillation counting. Palmitate oxidation was determined as the capture of ^14^CO_2_ (complete oxidation) and ^14^C-acid-soluble metabolites (ASMs), a measure of tricarboxylic acid (TCA) cycle intermediates and acetyl esters (incomplete oxidation)^68^, from palmitate ([1-^14^C] palmitate at 0.5 μCi/ml, 150 μM) in the presence and absence of cold pyruvate (5 mM), as described by Kim *et al*.^69^,^70^. Briefly, the reaction mixture contained 100 mM sucrose, 10 mM Tris/HCl, 80 mM KCl, 1 mM MgCl_2_, 2 mM L-carnitine, 0.1 mM malate, 2 mM ATP, 50 μM CoA, 1 mM dithiothreitol, 0.2 mM EDTA, and 0.5% BSA. Reactions were performed for 60 min at 30 °C after the addition of the enriched mitochondria fraction. One-hundred μl of 2 M sulfuric acid was added to terminate the reaction, and the CO_2_ produced during the incubation was trapped in 200 μl of 1 M NaOH. The acidified medium was stored at 4 °C overnight, and ASMs were then assayed in supernatants of the acid precipitate. Radioactivity of CO_2_ and ASMs was determined by liquid scintillation counting. PDH activity was assessed from 1 mM [1-^14^C] pyruvate in the presence or absence of 5mM L-carnitine and the ^14^CO_2_ released was quantified by scintillation counting.

### Statistics

Statistical significance of differences between the groups was determined by 2-way analysis of variance (ANOVA) followed by Bonferroni *post-hoc* test using Prism 5 software (GraphPad, CA, USA). The data are presented as means ± SD, and the level of significance was established *a priori* at *P* < 0.05.

## Additional Information

**How to cite this article**: Mucerino, S. *et al*. Alterations in the carnitine cycle in a mouse model of Rett syndrome. *Sci. Rep.* **7**, 41824; doi: 10.1038/srep41824 (2017).

**Publisher's note:** Springer Nature remains neutral with regard to jurisdictional claims in published maps and institutional affiliations.

## Supplementary Material

Supplementary Information

## Figures and Tables

**Table 1:** Electrophysiological values determined in 11-month-old WT and *Mecp2* ^+/−^ mice.

Group name	WT (n = 15)	*Mecp2*^+/−^ NQTc (n = 30)	*Mecp2*^+/−^ LQTc (n = 10)
RR (ms)	115.5 ± 3.5	116.3 ± 4.2	117.2 ± 3.9
QT (ms)	398 ± 12	401 ± 13	480 ± 14^***^
QTc (ms)	415.3 ± 2.4	418.0 ± 3.5	496.7 ± 3.3^***^

Data are expressed as mean ± SD. ^***^P < 0.001 *versus* age-matched WT and *Mecp2*^+/−^ NQTc.
