# Supplementary material for: Alterations in the carnitine cycle in a mouse model of Rett syndrome
Source: Sci Rep. 2017 Feb 2;7:41824. doi: 10.1038/srep41824 (PMC5288798; doi:10.1038/srep41824)
Supplement: Supplementary Information [file srep41824-s1.pdf]

**Supplementary Information**

# Alterations in the carnitine cycle in a mouse model of Rett syndrome

Sabrina Mucerino, Anna Di Salle, Nicola Alessio, Sabrina Margarucci, Raffaella Nicolai, Mariarosa A.B. Melone, Umberto Galderisi, Gianfranco Peluso

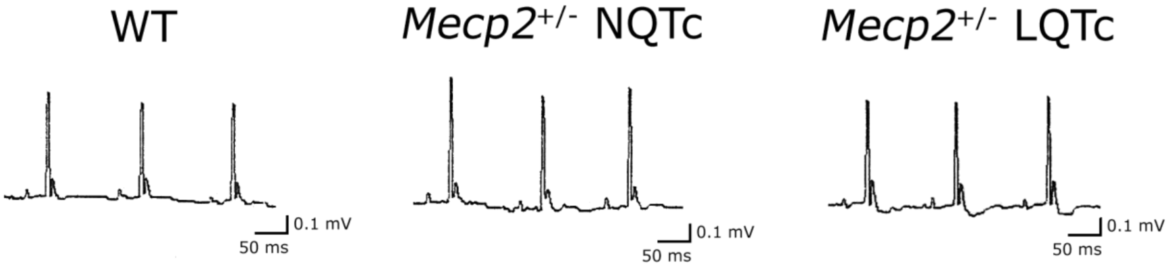


**Figure S1, Related to Table 1.** Representative ECGs from 11-month-old WT, *Mecp2*$^{+/-}$ NQTc and *Mecp2*$^{+/-}$ LQTc mice at baseline.

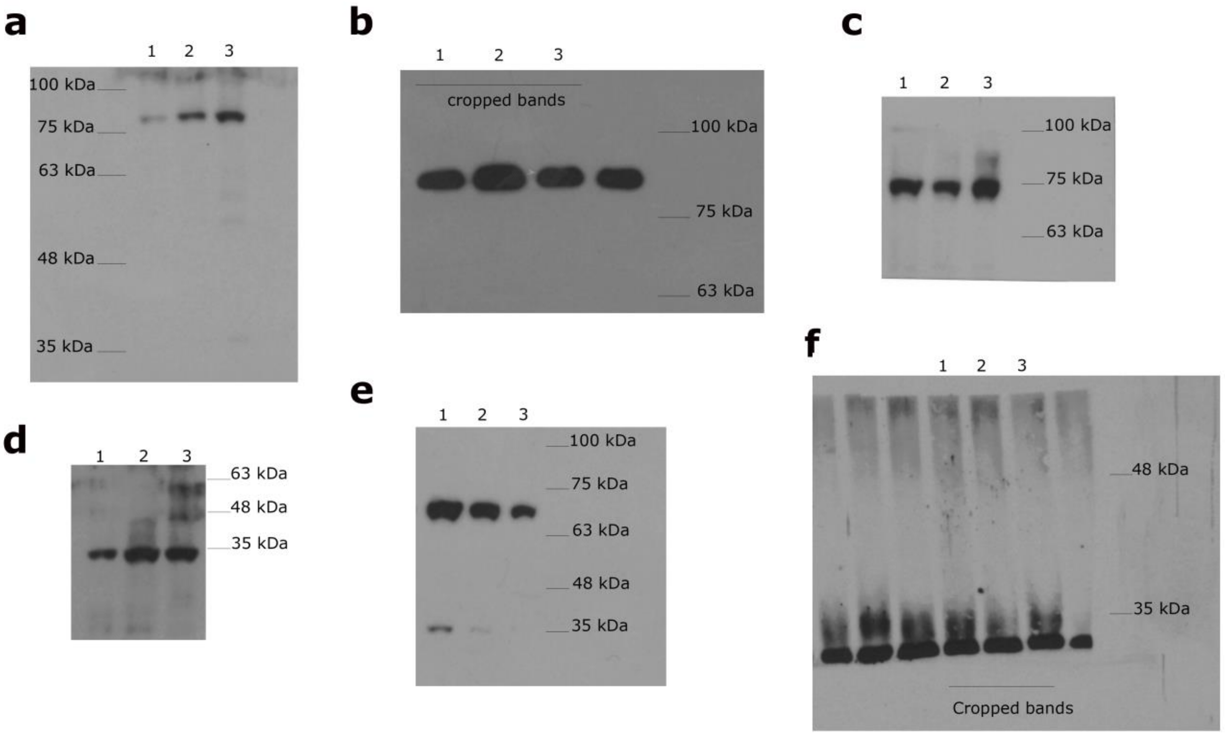


**Figure S2, Related to Figure 1.** Uncropped Western blots (see Fig. 1c) of (a): CPT1A detected using mouse polyclonal anti-carnitine palmitoyltransferase 1A; (b): CPT1B detected using polyclonal anti–carnitine palmitoyltransferase 1B; (c): CPT2 detected using monoclonal anti–carnitine palmitoyltransferase 2; (d): CACT detected using polyclonal anti-carnitine acylcarnitine translocase; (e): CrAT detected using polyclonal anti-carnitine acetylcarnitine transferase; (f): VDAC1 detected using polyclonal anti-VDAC1/Porin, as described in the Experimental section of this article. Legend: 1: WT; 2: *Mecp2*$^{+/-}$ NQTc; 3: *Mecp2*$^{+/-}$ LQTc.

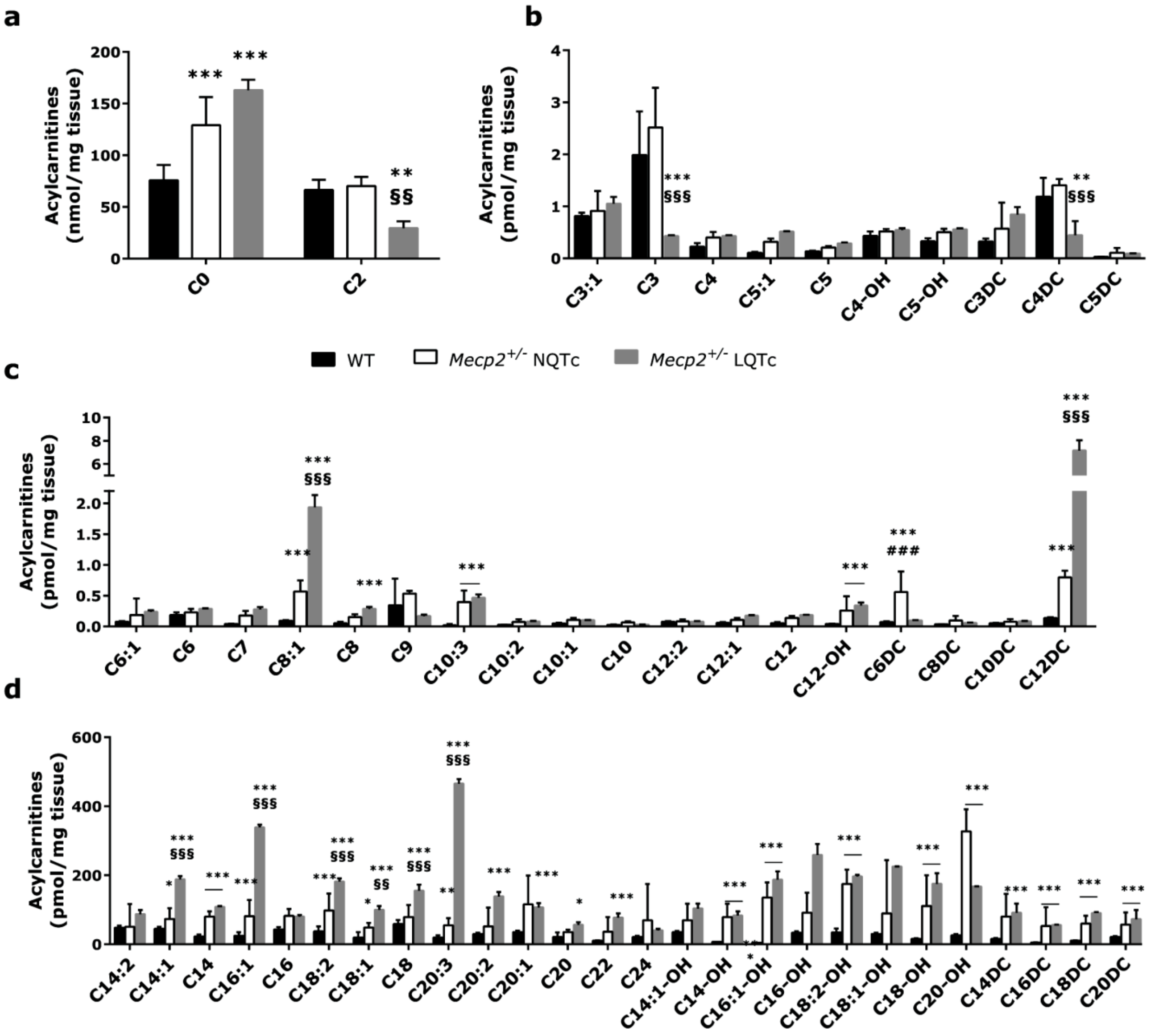


**Figure S3, Related to Figure 2.** Free carnitine and acylcarnitine profiling in heart tissues of WT (n=15), *Mecp2*$^{+/-}$ NQTc (n=30) and *Mecp2*$^{+/-}$ LQTc (n=10) mice. The acyl chain length (C) is denoted by the corresponding metabolite number (e.g., C0 = free carnitine, C2 = acetylcarnitine; C3 = propionylcarnitine). (a) C0 and C2 levels; (b) short-chain, (c) medium chain and (d) long-chain acylcarnitines. Data are expressed as mean ± SD. Statistically significant variations: *$P$<0.05 *versus* WT; **$P$<0.01 *versus* WT; ***$P$<0.001 *versus* WT; §§$P$<0.01 *versus* *Mecp2*$^{+/-}$ NQTc; §§§$P$<0.001 *versus* *Mecp2*$^{+/-}$ NQTc; ###$P$<0.001 *versus* *Mecp2*$^{+/-}$ LQTc.

**Insulin tolerance testing**

Ten-month-old mice were fasted for 4 hours and then injected intraperitoneally with 7.0 pmol/L. Blood glucose was measured immediately before the insulin challenge and 15, 30, 60 and 120 min thereafter by tail bleeds of approximately 10 µl per sample. Samples were analyzed with an Accu-Chek Aviva (Roche) blood glucometer.

**$O_2$ consumption/$CO_2$ expiration**

As a measure of metabolic function, we assessed gas exchange by measuring carbon dioxide ($CO_2$) production and oxygen ($O_2$) consumption as described by Schaevitz et al.[1]. Briefly, 10-month-old mice were placed in a cylindrical chamber with a fan to control chamber temperature. The chamber was connected on one side to an air pump and a flow meter, and on the other side to $CO_2$ and $O_2$ analyzers. Room air was supplied at 200 ml/min to the chamber by the pump, and gas concentrations of $CO_2$ and $O_2$ were measured. The experimental set-up was run without a mouse in the chamber to establish concentrations of $CO_2$ and $O_2$ in room air. The mouse was then placed in the chamber and gas concentrations were measured over a period of 20 min (10 min for habituation and 10 minutes to determine $CO_2$ and $O_2$ concentrations). After 20 min the mouse was returned to its home cage. Rates of $CO_2$ expiration and $O_2$ consumption were calculated by evaluating the difference between baseline gas concentration and concentrations while the mouse was in the chamber.

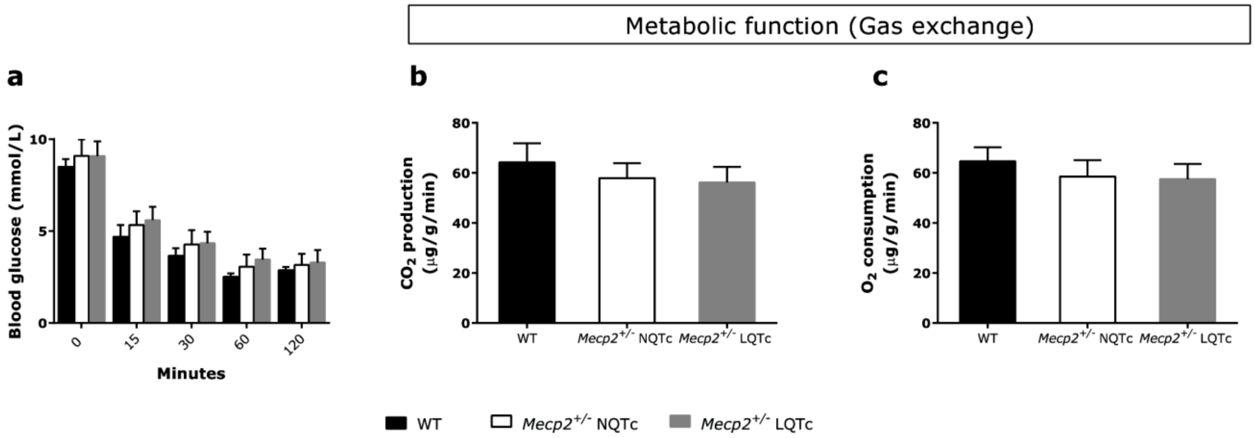


**Figure S4.** Metabolic analyses in WT (n=15), *Mecp2*$^{+/-}$ NQTc (n=30) and *Mecp2*$^{+/-}$ LQTc (n=10) mice. (a) Analysis of insulin tolerance. After fasting (t = 0 minutes) glucose levels did not differ between *Mecp2*$^{+/-}$ and WT mice. Similarly, the results of the insulin tolerance test did not differ among the groups of mice. Metabolic function (gas exchange) was assessed by measuring $CO_2$ production (b) and $O_2$ consumption (c). Although $CO_2$ and $O_2$ levels tended to be lower in RTT than in WT mice, the differences were not significant. Data are expressed as mean ± SD.

**Table S1 – Primers for qRT-PCR**

| GENE | FORWARD PRIMERS (5’-3’) | REVERSE PRIMERS (5’-3’) |
|---|---|---|
| *Cpt1A* | CCAGGCTACAGTGGGACATT | GAACTTGCCCATGTCCTTGT |
| *Cpt1B* | GGTCCCATAAGAAACAAGACCTC | AGACGATGTAAAGGGCAGAAGAGG |
| *Cpt2* | TCCTCGATCAAGATGGGAAC | GATCCTTCATCGGGAAGTCA |
| *Cact* | CCTGCCAGTGGGATGTATTT’ | TTTTCCAGGAGGTGCAGTCT |
| *Crat* | CAAGCAGGACTCAGTGGTGA | TTTCGGTGGTTGGAGGTTAG |
| *Actb* | GCTTCTTTGCAGCTCCTTCGT | CCAGCGCAGCGATATCG |
| *Hprt1* | ACCTTCTATGAATGTTACTG | GATAAGCGACAATCTACC |

**Table S2 – Body weight in 11-month-old WT and *Mecp2*$^{+/-}$ mice**

| | **WT (n=15)** | ***Mecp2*$^{+/-}$ NQTc (n=30)** | ***Mecp2*$^{+/-}$ LQTc (n=10)** |
|---|---|---|---|
| **Body weight (g)** | 24.68±3.75 | 32.64±5.59$^{**}$ | 30.12±4.39$^{**}$ |

Data are expressed as mean ± SD. $^{**}P$<0.01 *versus* WT.

**Table S3 – ATP level determined in explanted heart of 11-month-old WT and *Mecp2*$^{+/-}$ mice**

| | **WT (n=15)** | ***Mecp2*$^{+/-}$ NQTc (n=30)** | ***Mecp2*$^{+/-}$ LQTc (n=10)** |
|---|---|---|---|
| **ATP level (mmol/L)** | 715.01±73.58 | 689.25±66.54 | 691.02±71.02 |

Data are expressed as mean ± SD.

**Table S4 – Citrate synthase (CS) specific activity in the cardiac mitochondrial enriched fractions of 11-month-old WT and *Mecp2*$^{+/-}$ mice**

| | **WT (n=15)** | ***Mecp2*$^{+/-}$ NQTc (n=30)** | ***Mecp2*$^{+/-}$ LQTc (n=10)** |
|---|---|---|---|
| **CS activity (nM/min/mg protein)** | 112.13±9.46 | 108.66±8.85 | 110.52±9.21 |

Data are expressed as mean ± SD.

**References**

1 Schaevitz, L. R. *et al.* Acetyl-L-carnitine improves behavior and dendritic morphology in a mouse model of Rett syndrome. *PLoS One* **7**, e51586, doi:10.1371/journal.pone.0051586 (2012).
